# NIFTI: An evolutionary approach for finding number of clusters in microarray data

**DOI:** 10.1186/1471-2105-10-40

**Published:** 2009-01-30

**Authors:** Sudhakar Jonnalagadda, Rajagopalan Srinivasan

**Affiliations:** 1 Department of Chemical and Biomolecular Engineering, National University of Singapore, 10 Kent Ridge Crescent, 119260 Singapore

## Abstract

**Background:**

Clustering techniques are routinely used in gene expression data analysis to organize the massive data. Clustering techniques arrange a large number of genes or assays into a few clusters while maximizing the intra-cluster similarity and inter-cluster separation. While clustering of genes facilitates learning the functions of un-characterized genes using their association with known genes, clustering of assays reveals the disease stages and subtypes. Many clustering algorithms require the user to specify the number of clusters a priori. A wrong specification of number of clusters generally leads to either failure to detect novel clusters (disease subtypes) or unnecessary splitting of natural clusters.

**Results:**

We have developed a novel method to find the number of clusters in gene expression data. Our procedure evaluates different partitions (each with different number of clusters) from the clustering algorithm and finds the partition that best describes the data. In contrast to the existing methods that evaluate the partitions independently, our procedure considers the dynamic rearrangement of cluster members when a new cluster is added. Partition quality is measured based on a new index called Net InFormation Transfer Index (NIFTI) that measures the information change when an additional cluster is introduced. Information content of a partition increases when clusters do not intersect and decreases if they are not clearly separated. A partition with the highest Total Information Content (TIC) is selected as the optimal one. We illustrate our method using four publicly available microarray datasets.

**Conclusion:**

In all four case studies, the proposed method correctly identified the number of clusters and performs better than other well known methods. Our method also showed invariance to the clustering techniques.

## Background

Clustering is a statistical technique that partitions a large number of objects into a few clusters such that objects within the same cluster are more similar to each other than to the objects in other clusters. Clustering is widely used in gene expression data analysis to cluster genes and/or samples (assays) based on their similarity in expression patterns. Since gene clusters are often enriched with genes involving in common biological processes, identifying such clusters discloses potential roles of previously un-characterized genes and provides insights into gene regulation. Similarly, clustering of samples reveals different stages or subtypes of diseases such as cancer leading to development of customized diagnostic procedures and therapies.

Despite the widespread use of clustering algorithms in gene expression data analysis [1-6], selection of clustering parameters continues to be a challenge. In many cases, the optimal specification of number of clusters, *k*, is difficult especially if there is inadequate biological understanding of the system [7]. A suboptimal specification of number of clusters can generally result in misleading results – either all classes may not be identified or spurious classes may be generated [8]. While the correct number of clusters can be identified by visual inspection in some cases, in most gene expression datasets, the data dimensions are too high for effective visualization. Hence, methods that find the optimal number of clusters are essential.

Several methods have been proposed for finding the number of clusters in data. The popular methods evaluate the partition using a metric and optimize it as a function of number of clusters. Comprehensive reviews of these methods are available elsewhere [9-11]. Here we briefly describe some recent methods recommended for gene expression data analysis. Tibshirani et al. [12] proposed the gap statistic that measures the difference between within-cluster dispersion and its expected value under the null hypothesis. The *k *that maximizes the difference is selected. Since the gap statistic uses within-cluster sum of squares around the cluster means to evaluate the within-cluster dispersion, this method is suitable for compact, well separated clusters. Dudoit and Fridlyand [13] proposed a prediction based re-sampling method for finding the number of clusters. For each value of *k*, the original data is randomly divided into training and testing sets. The training data is used to build a predictor for predicting the class labels of the test set. The predicted class labels are compared to that obtained by clustering of test data using a similarity metric. This value is compared to that expected under an appropriate null distribution. The *k *for which the evidence of significance is the largest is selected. Ben-Hur et al. [14] proposed a similar re-sampling approach where two random subsets (possibly overlapping) are selected from the data. The two random subsets are subsequently clustered independently and the similarity between the resulting partitions is measured. The similarity is measured for multiple runs and its distribution is visualized for each *k*. The optimal number of clusters is selected where transition from high to low similarity occurs in the distribution. The approach of Dudoit and Fridlyand as well as Ben-Hur et al. assume that the sample subset can represent the inherent structure in the original data which may not be true for small clusters. Furthermore, the user has to manually locate the transition in Ben-Hur et al. approach.

**Figure 1:**
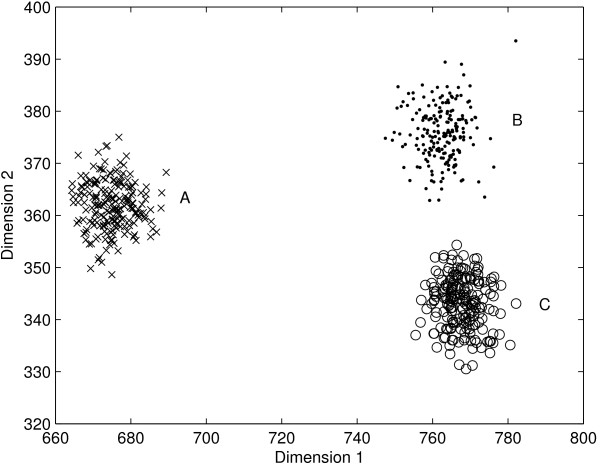
**Two dimensional artificial dataset with 3 inherent clusters (A, B, and C)**. Clusters B and C are closer to each other and far from Cluster A.

**Figure 2:**
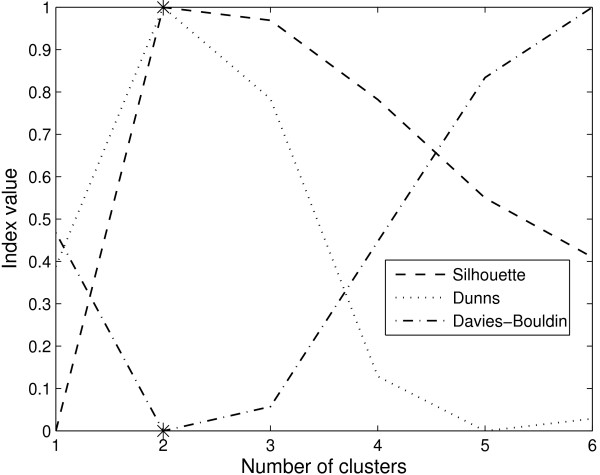
**Cluster validation results for the artificial dataset in Figure 1**. All three indices, Silhouette (dash line), Dunn's (dot line), and Davies-Bouldin (dash-dot line) incorrectly predict 2 clusters although the underlying data can be seen to have 3 clusters (* indicates the optimal number of clusters predicted by specific index).

Recently, Bolshakova and Azuaje [15] employed Silhouette [16], Generalized Dunn's index [8], and Davies-Bouldin index [17] on gene expression data. These methods use the intra- and inter-clusters distances to identify the best partition. In general, cluster validation is easier when the underlying clusters are well separated. But, most cluster validation methods lead to suboptimal results when inter- and intra-cluster distances vary largely. To illustrate this, consider the artificial dataset in Figure 1 consisting of 600 objects in three clusters (A, B, and C). Clusters B and C are closer to each other and far from Cluster A. Figure 2 shows the results of Silhouette, normalized Dunn's and normalized Davies-Bouldin indices for this dataset. For ease of visualization, all indices have been min-max re-scaled to [0 1]. For a given index value *I*_*k*_(*k *= 1,2,3, ... *k*_*max*_), the re-scaled index value is obtained as

(1) $${\hat{I}}_{k}=\frac{I_{k}-min(I_{k})}{max(I_{k})-min(I_{k})}$$

Silhouette, Generalized Dunn's index, and Davies-Bouldin indices incorrectly identified only 2 clusters in this dataset. A partition with two clusters {*A*} and {*B *∪ *C*} is more favorable according to intra- and inter-cluster distance based methods. Gene expression data contain clusters of different sizes, shapes, and there exist smaller clusters within the larger well-separated cluster [18]. Hence, the methods for finding number of clusters based on intra- and inter-cluster distances do not perform well for gene expression data (see results). This finding motivates development of new methods that do not rely on intra- and inter-cluster distances.

**Figure 3:**
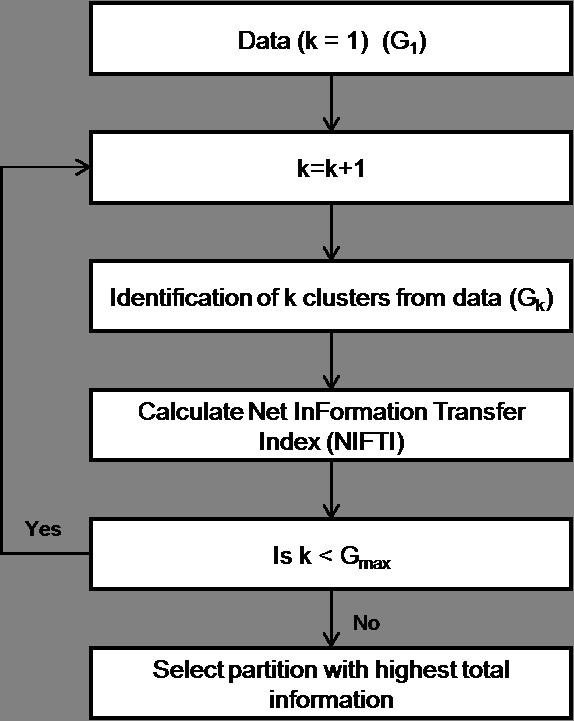
**Proposed cluster validation procedure**. The procedure starts with unclustered data (*G*_1_). In each subsequent generation, an additional cluster is added and the data reclustered. The Net InFormation Transfer calculated based on the evolution of objects during the generation. This procedure is carried out for a predefined number of generations (*G*_*max*_). Finally the partition with highest total information is selected as the optimal partition.

In this paper, we propose a new method to find optimal number of clusters in the data. Our approach is based on an evolutionary view of the clustering process (Figure 3). We start by considering the whole dataset as a single cluster and notate it as Generation 1 (*G*_1_). In each subsequent generation, the number of clusters, *k*, is incremented by one and the data re-clustered. A generation with *k *clusters is notated as *G*_*k*_. The net change in the information content due to the addition of a cluster is measured using Net InFormation Transfer Index (NIFTI). NIFTI includes two components – *direction *of information change and *magnitude *of information change – in its calculation. The *direction *of change indicates whether information is gained or lost during evolution. The *magnitude *indicates the extent of change. During evolution, objects from *i*^*th *^cluster, $C_{k}^{i}$, in the current generation, *G*_*k*_, will be distributed across several clusters in the next generation, *G*_*k*+1_. The clusters in *G*_*k*+1 _that receive objects from $C_{k}^{i}$ are called as offspring of parent cluster $C_{k}^{i}$. NIFTI considers this rearrangement of cluster members when a new cluster is added for calculating the information change. The net information change is the sum of the information change for all parent clusters. Information increases if offspring clusters are separable. We use a simple but effective procedure with statistical basis to check the separability of offspring clusters. The *magnitude *of information change is calculated using information theory. This evolutionary procedure is carried out for a predefined number of generations (*G*_*max*_). The Total Information Content, *TIC*, of a partition is defined as the cumulative information gained till that generation. A partition with the highest *TIC *is selected as the best partition. While testing for separability of clusters, NIFTI does not give weightage for largely separated clusters or penalize marginally separated clusters, thus eliminates the problems associates with varying inter-cluster distances.

## Results

Four publicly available microarray datasets are used to illustrate the performance of the proposed approach. The first two datasets are time-course datasets. In time-course datasets, genes are clustered based on their similarity in expression patterns. The other two datasets contain data from different samples.

Two different clustering techniques, namely k-means and model-based, are used for generating partition with different number of clusters. The distance metrics used for clustering are the same as those used by the data publishers *i.e *Pearson coefficient for first, third, and fourth case studies and standard correlation coefficient for the second dataset. In all the case studies, the maximum number of generations, *G*_*max *_is selected as [19]:

(2) $$G_{max}\leq \sqrt{N}$$

where *N *is the number of objects to be clustered.

### Case Study 1 : Yeast cell-cycle data

The Yeast cell-cycle dataset was generated by Cho et al. [20]. Oligonucleotide microarrays were used to monitor the expression levels of all known and predicted Yeast genes during two cell-cycles. Expression levels were measured at 17 time points with a time period of 10 min. The aim of this experiment was to identify the cell-cycle controlled genes in Yeast. Cho et al. visually observed the highly variant genes for consistent periodicity during the cell-cycle and identified 384 genes. These 384 genes were classified into five classes – early G1, late G1, S, G2, and M phases – based on their peak expression. Since the number of clusters is known for this dataset, the 384 cell-cycle genes are used to validate the proposed method.

**Figure 4:**
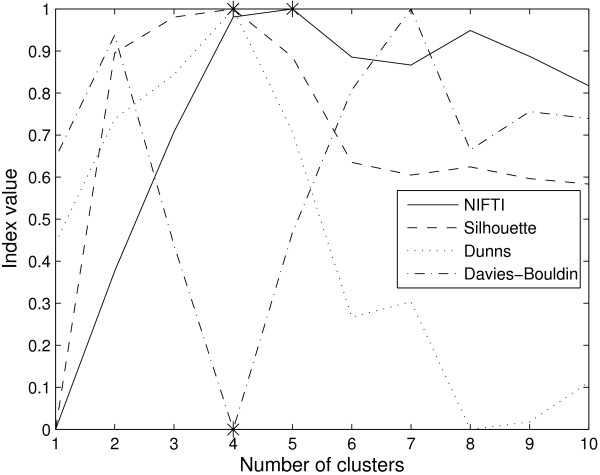
**Results for Yeast cell-cycle dataset using k-means clustering**. NIFTI (solid line) correctly finds 5 clusters in this dataset. Silhouette (dash line), Dunn's (dot line), and Davies-Bouldin (dash-dot line) indices predict only 4 clusters.

The proposed method, NIFTI, correctly identifies five clusters in this dataset using k-means method (Figure 4). For comparison, the results for Silhouette, Dunn's, and Davies-Bouldin indices are shown in Figure 4. All three indices predict 4 clusters in this data. The reason is as follows. At *k *= 4, genes from S and G2 phases are combined into one cluster while those from Early G1, Late G1, and M phases are clustered correctly. These four clusters are well-separated. When the number of clusters is increased to 5, while S and G2 clusters are identified correctly, the inter-cluster distance is small. The three methods therefore identify the partition with four clusters as optimal. In contrast to these distance based methods, the proposed method gives no weightage for larger inter-cluster distances and correctly identifies 5 clusters.

**Figure 5:**
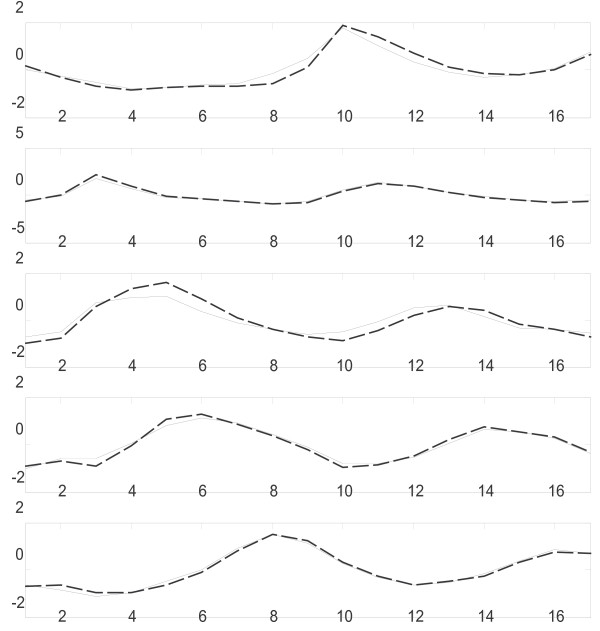
**Mean expression levels of Yeast cell-cycle clusters**. Solid line represents the mean expression profile of clusters reported by [20] and dash line corresponds to the optimal clusters from NIFTI. A strong similarity between the two can be observed.

The five clusters identified by k-means clustering correspond to the five phases of cell-cycle – early G1, late G1, S, G2, and M phases. For example, cluster 1 contains the cell-cycle regulated genes including PCL9, SIC1 and DNA replication genes CDC6 and CDC46 that are classified into early G1 by cho et al. [20]. The mean expression profile of this cluster shows single peak during the early stage of G1 (Figure 5). Similarly, other clusters are also enriched with genes that are classified into one of the reported clusters and their mean expression profiles peak during the corresponding stages (Figure 5).

**Figure 6:**
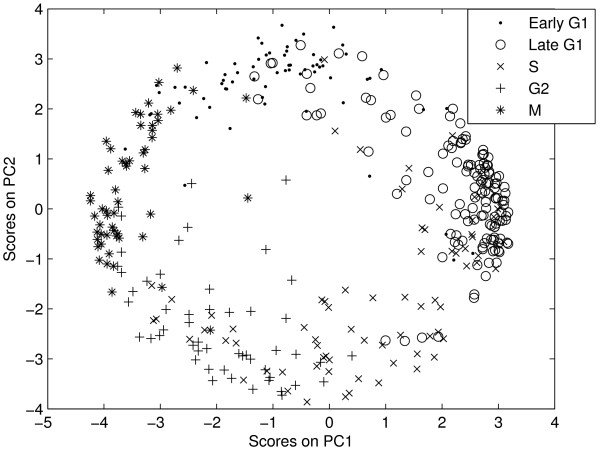
**Scores plot of Yeast cell-cycle dataset**. The first two PCs capture 65% variance.

However, some of the genes especially S phase genes are found to be 'mis-classified' by k-means clustering algorithm. To understand the discrepancy, we used Principal Component Analysis and plotted the scores with the first two dominant Principal Components (Figure 6). From Figure 6, it is clear that some of the genes from reported classes, especially S phase genes, are distributed to other classes. The k-means algorithm put those genes in appropriate classes which explains the mismatch between the reported and k-means partitions [see additional file 1].

**Figure 7:**
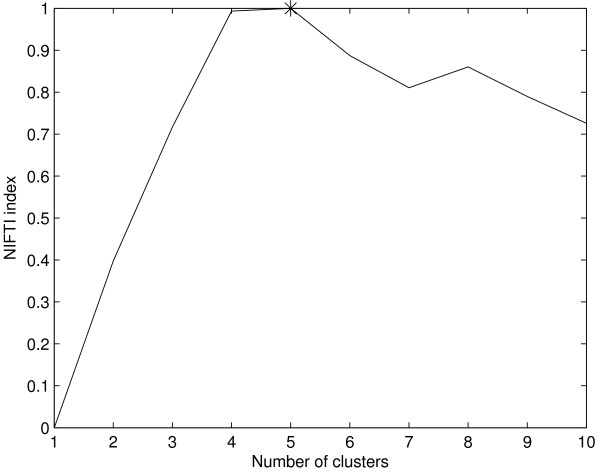
**Results for Yeast cell-cycle dataset using model-based clustering**. NIFTI correctly finds 5 clusters in this dataset.

**Figure 8:**
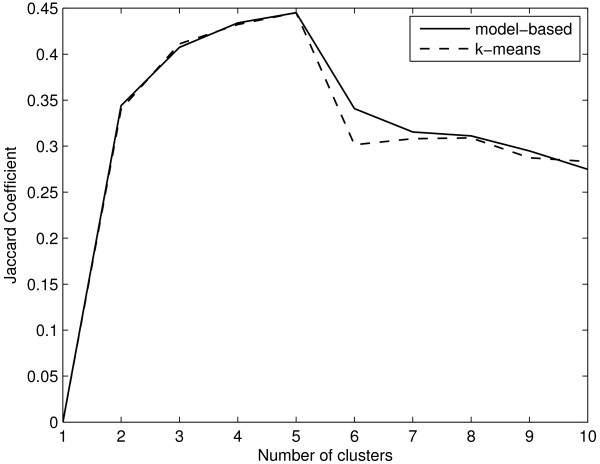
**Jaccard Coefficient for Yeast cell-cycle dataset**. The JC has a maximum at *k *= 5 indicating that there are 5 clusters.

Results for this dataset using model-based clustering are shown in Figure 7. NIFTI correctly identifies 5 clusters using model-based clustering as well. Since the 'true' (reported) partition is available for this dataset, we compare the clustering results using k-means and model-based clustering with reported partition using Jaccard Coefficient (JC) which measures the similarity between two partitions. Let *C *be the partition from the clustering algorithm and *P *be the reported solution. The JC measures the extent to which *C *matches with *P*

(3) $$JC=\frac{n_{11}}{n_{11}+n_{10}+n_{01}}$$

where *n*_11 _is the number of pairs of objects that are in the same cluster in both *C *and *P*, *n*_10 _is the number of pairs of objects that are in the same cluster in *C *but not in *P*, and *n*_01 _is the number of pairs of objects that are in the same cluster in *P *but not in *C*. JC takes a value between 0 (complete mismatch) and 1 (perfect match). The better the agreement between identified and the 'true' solution, the higher the value of JC. Figure 8 shows the JC for Yeast cell-cycle five phase criterion data as a function of number of clusters using k-means and model-based algorithm. The JC takes a maximum value of 0.445 at *k *= 5 indicating that in the given range of *k *the extracted partition best matches with the reported one. This clearly shows that the 5 clusters identified using proposed method are correct.

### Case Study 2 : Serum data

The Serum gene expression dataset is reported by Iyer et al. [21]. In this study, the response of human fibroblasts to serum was measured using microarrays containing around 8000 probes. Iyer et al. employed filtering techniques and shortlisted 517 most variant genes. They used hierarchical clustering and identified 10 clusters in this dataset using visualization tools. We use these 517 genes in this case study.

**Figure 9:**
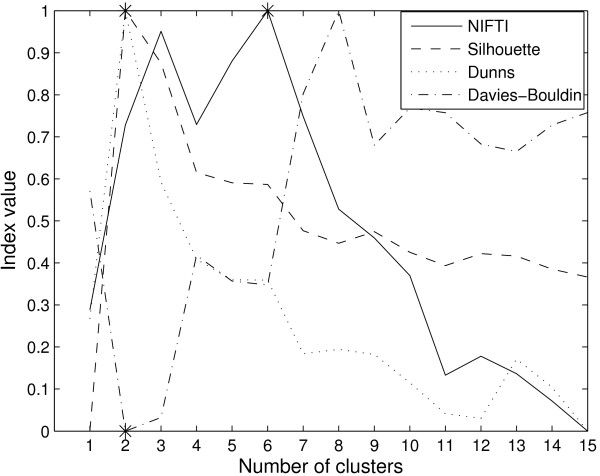
**Results for Serum dataset using k-means clustering**. NIFTI (solid line) predicts 6 clusters. Silhouette (dash line), Dunn's (dot line), and Davies-Bouldin (dash-dot line) estimate only 2 clusters.

**Figure 10:**
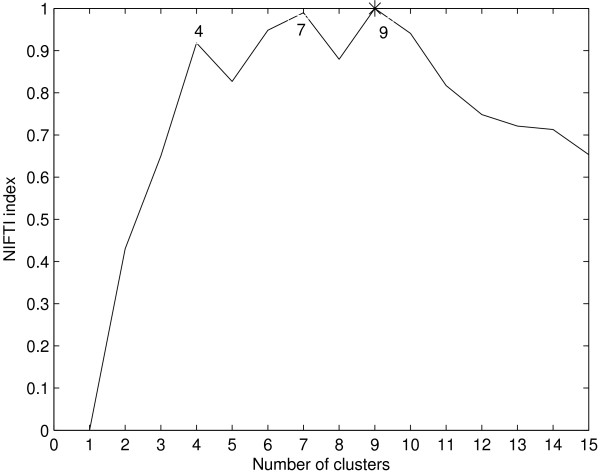
**Results for Serum dataset using model-based clustering**. NIFTI index has multiple peaks with a maximum peak ak *k *= 9. However, the Jaccard coefficient between the partition from model-based clustering and expert partition has maximum at *k *= 6 (Figure 11).

**Figure 11:**
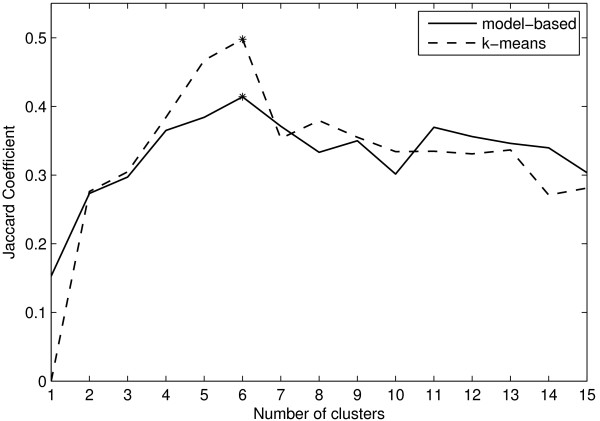
**Jaccard Coefficient for Serum dataset**. The Jaccard Coefficient for Serum dataset has maximum at number of clusters *k *= 6 indicating that identifying 6 clusters is correct.

NIFTI identifies 6 clusters in this dataset using k-means clustering (Figure 9). This result is supported by an other independent study using a graph-theoretical clustering algorithm [6]. The Silhouette, Dunn's and Davies-Bouldin indices identify only 2 clusters in the dataset (Figure 9). This dataset is more complex than the previous one. It contains two large clusters – one with up-regulated genes and another with down-regulated genes. All the other clusters are embedded in these large clusters. The ratio of difference between the intra- and inter-clusters distances is highest at *k *= 2. So any distance based method will generally identify only two clusters in this dataset. Multiple peaks were observed for NIFTI index for this dataset with the highest peak at *k *= 9 when model-based clustering is used for generation partitions (Figure 10). However, the Jaccard Coefficient between the partitions from model-based clustering and the reported partition has the highest value at *k *= 6 (Figure 11) indicating 6 clusters in this dataset.

In the next two case studies, the datasets contain gene expression data from different cancer samples. In these datasets, samples are clustered based on their similarity in expression patterns. Model-based clustering is not suitable for these datasets as it uses covariance matrix in its computation. The estimation of covariance matrix is inaccurate for sample clustering as the number of samples in each cluster are very small. So results are given for only k-means clustering.

### Case Study 3 : Lymphoma data

The lymphoma dataset was reported by Alizadeh et al. [22]. In this experiment, cDNA microarrays were used to characterize gene expression patterns in adult lymphoid malignancies. After filtering, the final data contain 4026 genes whose expression levels were measured using 96 arrays. The dataset comprises samples from three prevalent adult lymphoid malignancies – Diffuse Large B-cell Lymphoma (DLBCL), Follicular Lymphoma (FL), and Chronic Lymphocytic lymphoma (CLL). For comparison, the normal lymphocyte subpopulation under a variety of conditions is also included. The objective of the study was to identify if the presence of malignancy and its type can be identified from gene expression patterns. Alizadeh et al. used hierarchical clustering for clustering the samples and identified two distinct subtypes of DLBCL-Germinal Center B-like DLBCL and Activated B-like DLBCL.

**Figure 12:**
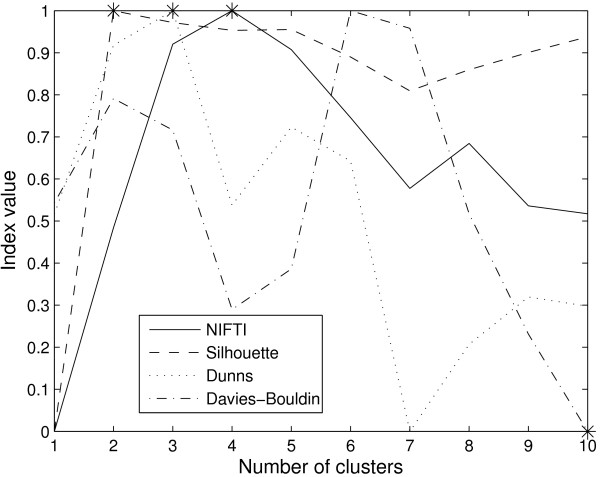
**Results for Lymphoma dataset**. NIFTI (solid line) finds 4 clusters in this dataset. Silhouette (dash line) identifies 2 clusters. Dunn's (dot line) predicts 3 clusters. Davies-Bouldin (dash-dot line) predicts 4 clusters.

NIFTI finds 4 clusters in this dataset using k-means clustering algorithm with Pearson correlation as the distance measure (Figure 12). Not surprisingly, these four clusters correspond to the four distinct branches of the dendrogram reported in [22]. Two of these clusters contain the samples from two subtypes of DLBCL namely germinal center B-like DLBCL and activated B-like DLBCL. The third cluster contains all FL and CLL samples along with the resting blood samples. Most of the cell-cycle control genes, checkpoint genes and DNA synthesis genes that are defined as 'proliferation signature' by [22] are under expressed in these samples. This makes these samples distinct from DLBCL samples in which the proliferation signature genes are up-regulated. The fourth cluster comprises the remaining normal lymphocyte subpopulation under different activation conditions. However, the transformed cell line samples which are grouped with other normal sub-populations by [22] are clustered with DLBCL samples by k-means. The over-expression of proliferation signature genes in these samples might be the reason that they appear 'closer' to DLBCL samples to k-means. Nevertheless, k-means clustering correctly clustered two out of the three DLBCL samples that were incorrectly clustered by the hierarchical clustering.

The Silhouette, Dunn's and Davies-Bouldin indices for this dataset are also shown in Figure 12. The Silhouette index estimates only 2 clusters and Dunn's index predicts 3. The lowest value of Davies-Bouldin index occurred at *k *= 10 in the range of *k *values tested (it continued to decrease further with increase of *k*). However, Davies-Bouldin index has a local minima at *k *= 4 indicating four clusters in this dataset. At *k *= 2, all DLBCL samples are grouped into one cluster and all other samples (FL, CLL, and normal) are lumped into other. At *k *= 3, the latter is split and normal samples are identified as the third cluster. This indicates that at *k *= 2 and *k *= 3 subclasses of DLBCL cannot be identified. Only at *k *= 4, the two subclasses of DLBCL are identified. This clearly shows the usefulness of proposed method to identify correct number of clusters that aids discovering novel sub-types of diseases.

### Case Study 4 : Pancreas data

The Pancreas dataset used in this study was reported by [23]. In this study, cDNA microarrays were used to analyze gene expression patterns in 14 pancreatic cell lines, 17 resected infiltrating pancreatic cancer tissues (two sub types), and 5 normal pancreases. The final filtered dataset consists of 1493 genes and 36 samples.

**Figure 13:**
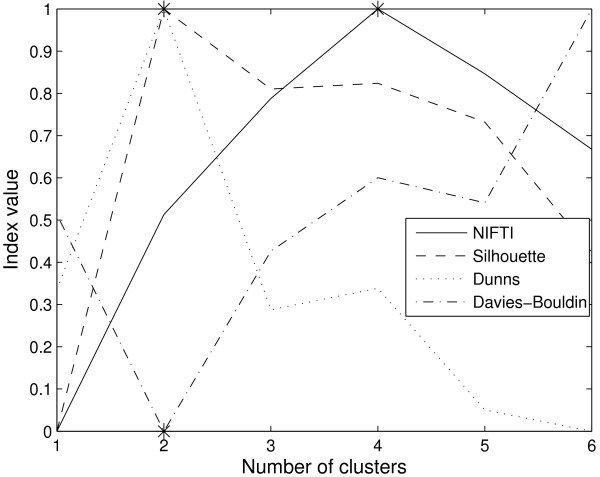
**Results for Pancreas dataset**. NIFTI (solid line) finds 4 clusters in this dataset. Silhouette (dash line), Dunn's (dot line), and Davies-Bouldin (dash-dot line) indices predict only 2 clusters.

As shown in Figure 13, Silhouette, Dunn's, and Davies-Bouldin indices estimate 2 clusters for this dataset. A partition with two clusters lumps together the normal and pancreatic cancer tissues into a single cluster. The second cluster contains all the pancreatic cancer cell lines. NIFTI estimates four clusters in this data. A partition with four clusters describes this data well: all cancer cell line samples are accurately placed in one cluster, all normal samples are grouped together, and two different cancer tissues are well separated into two clusters. Only one sample was found to be mis-clustered. This partition with four clusters also exactly matches the dendrogram reported in [23].

## Discussion and Conclusion

The use of clustering techniques in gene expression data analysis is increasing rapidly. To obtain the best results from these clustering techniques, optimal specification of the number of clusters is essential. Hence, methods that automatically identify the number of clusters in high-dimensional gene expression data have been proposed. Methods for finding the number of clusters in a dataset can be classified as global or local methods [24]. Global methods evaluate clustering results by calculating some measure over the entire dataset whereas local methods consider pairs of clusters and test whether they should be amalgamated. The disadvantage of the global methods is that there is no definition for the measure for *k *= 1, *i.e*., the global methods do not provide any clue whether the data should be clustered or not. Since local methods consider pairs of clusters, they can be used to decide if data should be clustered. The disadvantage of local methods is that they need a threshold value or significance level to decide whether the clusters should be amalgamated. The proposed approach combines both local and global approaches. At the local level, offspring clusters are checked for overlap and this information is converted into a global index.

The well-known methods for finding the number of clusters use within-cluster dispersion and/or inter-cluster distances. These 'distance' based methods are generally suitable when clusters are compact and well-separated but fail when sub-clusters exist. Our approach overcomes this limitation by giving no extra weightage for larger inter-cluster distances. In our approach, clusters lose or gain information based on intersection with other clusters. The actual distance between the clusters is not taken into consideration. Furthermore, the cumulative way of measuring information content of a partition ensures that information increase as long as a non-intersecting cluster can be identified.

We have compared the performance NIFTI with four other methods – Silhouette, Dunn's, Davies-Bouldin, and Gap statistic – in terms of percentage of correct prediction of actual number of clusters in artificial datasets. The synthetic datasets are generated with number of dimensions *d *= 2, 3 and 5 and number of clusters *k *= 3, 5, and 8. For each combination of *d *and *k*, 100 artificial datasets are generated and k-means clustering is used for generation of partitions. The results are given in additional file 2. For a given *d*, the performance of Silhouette, Dunns and Davies-Bouldin indices decreased significantly with increasing *k*. For example, for 2-dimensional datasets, the percentage success of these methods dropped from 70% to 20% as *k *increased from 2 to 8. This is mainly due to decrease in inter-cluster distance with increase in number of clusters. Similar trend of decreasing performance is observed with Gap statistic as well. Also, its performance is very poor (< 20%) with large number of clusters. In all the case studies, NIFTI performed better compared to the other methods. The performance of NIFTI is largely independent of the number of clusters and number of dimensions. This study clearly indicates the efficacy of NIFTI in predicting the number of clusters in data.

However, the proposed method has a limitation. It models clusters as hyper-spheres. Even though modeling clusters as hyper-spheres simplifies the task of finding cluster intersections, it may lead to incorrect results in case the clusters do not have a spherical shape. Nevertheless, this procedure consistently identified the 'correct' number of clusters suggesting, in part, the spherical shape of gene clusters. An independent study also reported that normalization techniques used in gene expression data analysis make the clusters spherical [4].

In this paper, the proposed method is evaluated using k-means clustering algorithm with Pearson correlation as distance measure for the Yeast cell-cycle and lymphoma datasets. The standard correlation coefficient (dot product of normalized vectors) is used for the Serum dataset. These two measures are bounded: the minimum and maximum distances are 0 and 2 respectively. On the other hand metrics such as Euclidian distance and Manhattan distance are unbounded. Hence, the affect of outliers will be high while estimating the cluster radii. This may lead to incorrect estimation of number of clusters. This can be overcome by suitable normalizing the data or selecting other ways to find cluster radius that are less sensitive to outliers. Further study using various distance metrics and clustering techniques is needed to further evaluate the method.

Generally computational time is an important issue in determining the number of clusters. In this study, we used 100 replicates of k-means algorithm for all datasets. The time required for finding number of clusters is less than 10 minutes for all datasets on a *Pentium *4 with 2.8 GHz processor.

## Methods

**Figure 14:**
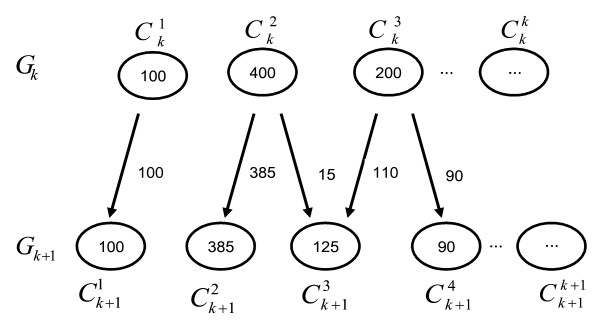
**Behavior of cluster members during evolution**. A few clusters in *G*_*k *_continue as single clusters in *G*_*k*+1 _while others disassociate or undergo leakage.

Let *Z*_*Nxm *_be the dataset to be clustered containing *N *objects on which *m *features are measured. In gene expression data analysis, *N *is number of genes and *m *is number of assays. We use a clustering algorithm to generate a series of partitions from *G*_1 _through *G*_*max *_with an increment of one cluster in each generation. The migration of the objects during evolution from parent clusters in *G*_*k *_to their offspring in *G*_*k*+1 _forms the basis for evaluating the quality of partition in *G*_*k*+1_. Consider the migration of objects among clusters during evolution from *G*_*k *_to *G*_*k*+1 _shown in Figure 14. Three scenarios are possible during evolution:

1. All objects in $C_{k}^{i}$ may continue to be clustered together as a single cluster in *G*_*k*+1_. We call this phenomenon as *cluster conservation*. Example: The cluster $C_{k}^{1}$ is conserved as $C_{k+1}^{1}$ with all objects intact.

2. Most members of $C_{k}^{i}$ may stay together as a single cluster in *G*_*k*+1_, but a few escape to other clusters. This phenomenon is termed as *cluster leakage*. Example: Out of 400 objects in cluster $C_{k}^{2}$ most stay together in $C_{k+1}^{2}$, 15 leak to $C_{k+1}^{3}$

3. Members of $C_{k}^{i}$ migrate to a small number ≥ 2 of clusters in *G*_*k*+1 _such that each recipient cluster receives a significant fraction of objects. This is called as *cluster disassociation*. Example: Cluster $C_{k}^{3}$ disassociates to $C_{k+1}^{3}$ and $C_{k+1}^{4}$

During evolution from *G*_*k *_to *G*_*k*+1_, some clusters are conserved, some disassociated, and others undergo leakage. The quality of the partition is measured in terms information transferred from *G*_*k *_to *G*_*k*+1 _using the Net InFormation Transfer Index (NIFTI). The TIC of partition is calculated for each generation as the sum of cumulative information transferred till that generation. The partition with the largest TIC is selected as the optimal one. The TIC for a partition at (*k *+ 1)^*th *^generation is given by:

(4) $$TIC_{k+1}=TIC_{k}+NIFTI_{G_{k}\to G_{k+1}}$$

where *TIC*_1 _= 0.

The optimal number of clusters is given by:

(5) $$k_{optimal}=\underset{1\leq k\leq k_{max}}{\arg\max}TIC_{k}$$

### Net InFormation Transfer Index (NIFTI)

The Net InFormation Transfer Index during evolution from *G*_*k *_to *G*_*k*+1 _is defined as the sum of the information changes of all parent clusters weighted by the fraction of total objects they contain.

(6) $$NIFTI_{G_{k}\to G_{k+1}}=\sum_{i}^{k}\frac{N_{k}^{i}}{N}\times g_{k}^{i}$$

where $N_{k}^{i}$ is the number of objects in *i*^*th *^parent cluster and $g_{k}^{i}$ is its change in information as it evolves from *G*_*k *_to *G*_*k*+1_. Equation 6 is similar to the one used by Li et al. [25] for calculating the information content of a partition.

The change in information of a parent cluster $C_{k}^{i}$ is given by:

(7) $$g_{k}^{i}=D_{k}^{i}\times M_{k}^{i}$$

$D_{k}^{i}$ is the direction (gain or loss) and $M_{k}^{i}$ the magnitude of information change arising from *i*^*th *^parent cluster.

The objective of clustering is to identify clusters where objects within a cluster are more similar to each other compared to objects within other clusters. Geometrically, this means that clusters should be distant and separable from each other in the *m *dimensional feature space. Here, we propose a statistical test to check whether offspring clusters are separable or not. If the offspring of parent cluster are separable from other sibling, information is deemed to have been gained during transfer and $D_{k}^{i}$ takes +1. In contrast, if offspring are not separable, information is deemed to be lost during transfer and $D_{k}^{i}$ is -1. In contrast to other methods, the NIFTI is not weighted as per the inter- and intra-cluster distances.

The magnitude of information change, $M_{k}^{i}$, is calculated using Shannon entropy given by:

(8) $$M_{k}^{i}=\sum_{j=1}^{r}-p_{k}^{ij}\ln p_{k}^{ij}$$

where *r *is the number of offspring and *p*^*ij *^(*j *= 1,2, ..., *r*) is the fraction of objects that *j*^*th *^offspring inherits.

As described before, during evolution from *G*_*k *_to *G*_*k*+1_, some clusters are conserved, some disassociated, and others undergo leakage. Consequently $M_{k}^{i}$ is 0 for conservation, small for leakage, and large for cluster disassociation. Offspring clusters are tested using a separability test and NIFTI increases if they are separable and decreases otherwise. We propose a simple but effective test for separability of clusters. The cluster separability test is described below.

### Test for separability of offspring

Though a parent cluster can result in many offspring, in practice it is observed that most members of a parent cluster migrate to a few proximal offspring. This is not a surprise since only one additional cluster is added at each step. Therefore, the incremental reorganization that takes place during evolution is minimal. We term those offspring which inherit large fractions of objects from a parent as the dominant offspring. The information transferred for a parent cluster can be approximated by considering only the dominant offspring. The information change arising from the other offspring (non-dominated) is very small and can be neglected. Hence, *r *in Equation 8 is set to 2 for all parent clusters.

Let *X *and *Y *be the two dominant offspring of a parent cluster given by:

(9) $$X=\underset{j}{\arg\max}p^{ij}$$

(10) $$Y=\underset{j\neq X}{\arg\max}p^{ij}$$

where *p*^*ij *^is the fraction of objects migrated from *i*^*th *^parent cluster, $C_{k}^{i}$ to the *j*^*th *^offspring cluster, $C_{k+1}^{j}$.

We use inter- and intra-cluster distances to identify whether *X *and *Y *are separable or not. *X *and *Y *are said to be separable if the distance between their centroid, *δ*_*XY*_, is larger than the sum of their radii (Δ_*X *_and Δ_*Y*_). A variety of methods can be used to measure the cluster radius [8]. Here, the mean distance between the cluster centroid to all members of that cluster is used for this purpose.

Radius of cluster *X*:

(11) $$\Delta_{X}=\frac{1}{\left|X\right|}\sum_{x\in X}d(x,{\bar{v}}_{X})$$

where |*X*| is the number of objects in *X*, *x *represents the object in cluster *X*, *d *is the distance metric used for clustering, and ${\bar{v}}_{X}$ the centroid of the cluster. Similarly the radius of cluster *Y *is given by:

(12) $$\Delta_{Y}=\frac{1}{\left|Y\right|}\sum_{x\in Y}d(x,{\bar{v}}_{Y})$$

The centroid distance between *X *and *Y *is the distance between their centroids given as:

(13) $$\delta_{XY}=d({\bar{v}}_{X},{\bar{v}}_{Y})$$

Hence, the separability of offspring of $C_{k}^{i}$ notated as $D_{k}^{i}$ is given by:

(14) $$D_{k}^{i}=\{\begin{array}{lll} +1 & if & \delta_{XY}\geq (\Delta_{X}+\Delta_{Y})\\ -1 & if & \delta_{XY}<(\Delta_{X}+\Delta_{Y}) \end{array}$$

Geometrically, the proposed procedure for finding the separability of clusters is equal to modeling each offspring clusters as a hyper-spheres with radii (Δ_*X *_and Δ_*Y*_)and check whether the hyper-spheres overlap. Statistically, this procedure is a hypothesis test with the following null and alternative hypotheses:

*H*_0 _= Offspring clusters are part of single cluster

*H*_1 _= Offspring clusters are not part of single cluster (*i.e*. different clusters)

The equations for hypothesis testing are derived considering the situation where a single cluster is artificially broken into two clusters. Let us consider a single cluster *C *containing *n *objects. Assume that the data is drawn from Gaussian distribution with mean *μ *and covariance matrix Σ. Without loss of generality, we can assume that the mean is at origin and covariance matrix has only diagonal elements and off-diagonal elements are all zero (if the original covariance matrix contains non-zero off diagonal elements it can be converted to diagonal matrix by principal axis rotation). Suppose, now that we partition *C *into two clusters (offspring), we can reject the null hypothesis using the distribution functions of both centroid distance and radii of offspring clusters. There are two cases:

1. Same variance in all dimensions *i.e*.

$$\Sigma =\left|\begin{array}{cccc} \sigma_{1}^{2} & 0 & \ldots & 0\\ 0 & \sigma_{2}^{2} & \ldots & 0\\ \vdots & \vdots & \ddots & \ldots \\ 0 & 0 & \ldots & \sigma_{m}^{2} \end{array}\right|$$

and $\sigma_{1}^{2}=\sigma_{2}^{2}=\ldots =\sigma_{m}^{2}$.

2. The *σ*_*^2^i*_s of Σ are different.

We derive the equations for proposed test of separability of offspring for case 1 and show how it can be extended to case 2.

#### Case 1

**Figure 15:**
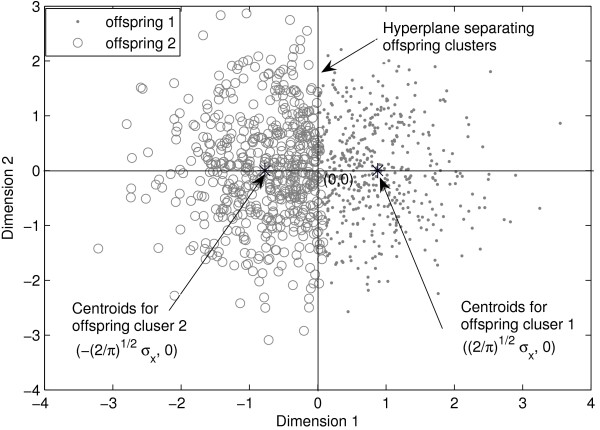
**Artificial partitioning of natural cluster**. A single natural cluster drawn from Gaussian distribution with mean at origin and identity covariance matrix. k-means clustering partitions this single cluster into two clusters separated by hyperplane.

Geometrically, this means that the cluster of objects form a spheroid in *m*-dimensional space. Application of any clustering algorithm to partition this cluster into two offspring results in optimal (based on the objective function used for clustering) partition. If we know the analytical solution for that optimal partitioning, we could determine the distribution functions for centroid distance and radii of clusters. Lacking the analytical solution for the optimal partitioning, we cannot derive the actual sampling distributions. However, approximate estimates can be obtained by considering the suboptimal partition provided by a hyperplane through the centroid of parent cluster [26]. This hyperplane approximation is schematically described in Figure 15 for two dimensional data. The data contains 1000 samples drawn from 2 dimensional Gaussian distribution with mean at origin and covariance matrix [1 0;0 1]. k-means clustering algorithm is used to generate the two partitions.

Because of the hyperplane, the centroids for individual offspring clusters will be same as centroid of original parent cluster except in one dimension (the dimension ⊥ to hyperplane). Let the dimension ⊥ to hyperplane is denoted as *f*. Then *f *follows half-normal distribution with mean $\sqrt{2/\pi \sigma }$ (Figure 15). So, the centroid distance between the two offspring is $2\sqrt{2/\pi \sigma }$. Considering the sample size, *n*, the squared centroid distance between the two offspring cluster follows Gaussian distribution with mean as ((*n*-1)/*n*)8/*πσ*^2 ^and variance 2((*n*-1)/*n*^2^)(64/*π*^2^)*σ*^4^. The squared radius of cluster Δ^2 ^also follows a Gaussian distribution with mean ((*m*-2)/*π*)*σ*^2 ^and variance 4((*m*- 8)/*π*^2^)*σ*^4 ^[26].

Now consider the Equation 14 for testing the separability of offspring clusters.

(15) *δ*_*XY *_≥ (Δ_*X *_+ Δ_*Y*_)

Squaring both sides

(16) $$\delta_{XY}^{2}\geq {\left(\Delta_{X}+\Delta_{Y}\right)}^{2}$$

Since the clusters are separated by a hyperplane passing through the origin, the two offspring clusters approximately contain same number of samples and hence (Δ_*X *_≈ Δ_*Y *_= Δ).

Hence the test of separability of offspring clusters reduces to

(17) *δ*^2 ^≥ 4 × Δ^2^

where the subscripts *X *and *Y *have been removed for convenience. Hence, the offspring clusters are deemed to be separable if

(18) *h *≥ 0

where *h *= *δ*^2 ^- 4 × Δ^2^

Using the distributions for *δ*^2 ^and Δ^2 ^derived above the distribution for above equations can be obtained. This distribution refers to the null distribution for the proposed hypothesis test as this derivation is through artificial portioning of a single cluster. Hence, the null hypothesis can be rejected considering the distribution of above equation. Since, both *δ*^2 ^and Δ^2 ^follows Gaussian distribution, *h *follows a Gaussian distribution with mean as $4\frac{\left(n-1\right)}{4}(4/\pi -m)\sigma^{2}$ and variance $\frac{2}{n}\left[\frac{64}{\pi^{2}}+8\left(m-8/\pi^{2}\right)\right]\sigma^{4}$.

The false discovery rate for rejecting the single cluster hypothesis can be calculated using the distribution of *h*. The false discovery rate is the probability of *h *> 0. The false discovery rate indicates the probability that a offspring of a single parent cluster are incorrectly deemed as two separable clusters. Table 1 shows the false discovery rate for different sample sizes, *n*, and number of dimensions, *m*. The values given in parenthesis are the false discovery rates obtained by computational study with 1000 datasets with mean at origin and *σ*^2 ^= 2. The false discovery rates are very low even for small samples sizes. It clearly shows that the proposed cluster separability test able to correctly identify the artificial break of natural clusters. When a natural clusters is artificially broken, NIFTI decreases. So, selecting a partition with highest NIFTI gives number of natural clusters in the data.

**Table 1:** False discovery rate of cluster separability test

Sample size(n)	m = 2	m = 3	m = 4	m = 5
25	0.0068 (0.199)	8.53E-7 (0.021)	2.99E-11 (0.001)	6.66E-16 (0.002)
50	1.84E-4 (0.008)	2.44E-12 (0.002)	0 (0)	0 (0)
100	1.81E-7 (0)	0 (0)	0 (0)	0 (0)

#### Case 2

Geometrically this means that the cluster form a ellipsoid in *m*-dimensional space. An Analytical solution is difficult for this case. However, it is possible to show that *δ*^2 ^- 4Δ^2 ^≥ 0 for many situations. Assuming that the hyperplane separating the two offspring cluster is ⊥ to the dimension of largest variance, the *δ*^2 ^is given by: $8/\pi \sigma_{max}^{2}$. Similarly, Δ^2 ^is given $\sum_{i=1,i\neq j}^{m}\sigma_{i}^{2}+(1-2/\pi )\sigma_{max}^{2}$ where *j *corresponds to the dimension of largest variance. Hence, the separability test *δ*^2^-4Δ^2 ^is given by: $4\sigma_{max}^{2}[4/\pi -1]-\sum_{i=1,i\neq j}^{m}\sigma_{i}^{2}$. This means the artificial partition of single cluster is detected by proposed separability criteria whenever the sum of variances in all directions (except the variance of largest direction) has value at least 0.275 × $\sigma_{max}^{2}$. Since this criteria is satisfied in most of the cases, the proposed test for separability works well even in this case. To check the performance of proposed separability test, we generated 1000 random datasets with 1000 samples each in 3-dimensional space with the largest variance as $\sigma_{max}^{2}$ = 3 and other variances are 0.75. In all the datasets the proposed method correctly identified the partition of a single cluster.

## Authors' contributions

Both SJ and RS contributed to the concept and methodology development. SJ implemented the methodology and conducted the data analysis and biological interpretation. RS supervised the study and assisted in implementation. SJ drafted the manuscript. Both authors read and approved the final manuscript.

## Supplementary Material

Additional File 1 **Scores plot of k-means results for Yeast cell-cycle dataset. **The first two PCs capture 65% variance. All the five clusters are homogenous and distinct. Click here for file

Additional File 2 **Supplementary Material.** Comparison of methods for finding number of clusters using artificial datasets. Click here for file
