# Supplementary figures and images for: NIFTI: An evolutionary approach for finding number of clusters in microarray data
Source: BMC Bioinformatics. 2009 Jan 30;10:40. doi: 10.1186/1471-2105-10-40 (PMC2669482; doi:10.1186/1471-2105-10-40)

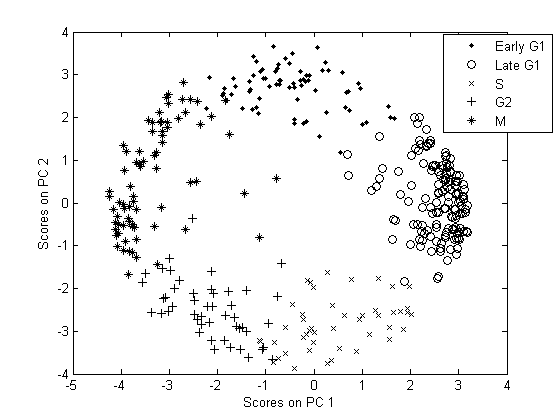

Supplement: Additional File 1 — Scores plot of k-means results for Yeast cell-cycle dataset. The first two PCs capture 65% variance. All the five clusters are homogenous and distinct. [file 1471-2105-10-40-S1.png]
