# Supplementary material for: NIFTI: An evolutionary approach for finding number of clusters in microarray data
Source: BMC Bioinformatics. 2009 Jan 30;10:40. doi: 10.1186/1471-2105-10-40 (PMC2669482; doi:10.1186/1471-2105-10-40)
Supplement: Additional File 2 — Supplementary Material. Comparison of methods for finding number of clusters using artificial datasets. [file 1471-2105-10-40-S2.doc]

Supplementary Material

**Comparison of methods for finding number of clusters using artificial datasets**

We have compared the performance of our method, NIFTI, with four other methods—Silhouette, Dunns, Davies-Bouldin, and Gap statistic—recommended for finding number of clusters in gene expression data. The performance of these methods is compared in terms of percentage of correct predictions of actual number of clusters in artificial datasets. The synthetic datasets are generated with number of dimensions *d* = 2, 3 and 5 and number of clusters *k =* 3, 5, and 8. For each combination of *d* and *k*, 100 artificial datasets are generated and k-means clustering is used for generation of partitions. The results are shown below:

For a given *d,* the performance of Silhouette, Dunns and Davies-Bouldin indices decreased significantly with increasing *k*. For example, for 2-dimensional datasets, the percentage success of these methods dropped from ~70% to ~20% as *k* increased from 2 to 8. This is mainly due to decrease in inter-cluster distance with increase in number of clusters. Similar trend of decreasing performance is observed with Gap statistic as well. Also, its performance is very poor (< 20%) with large number of clusters. In all the case studies, NIFTI performed better compared to the other methods. The performance of NIFTI is largely independent of the number of clusters and number of dimensions. This study clearly indicates the efficacy of NIFTI in predicting the number of clusters in data.
